# FoxM1 is an independent poor prognostic marker and therapeutic target for advanced Middle Eastern breast cancer

**DOI:** 10.18632/oncotarget.24739

**Published:** 2018-04-03

**Authors:** Abdul Khalid Siraj, Poyil Pratheeshkumar, Sandeep Kumar Parvathareddy, Zeeshan Qadri, Saravanan Thangavel, Saeeda Ahmed, Fouad Al-Dayel, Asma Tulbah, Dahish Ajarim, Khawla S. Al-Kuraya

**Affiliations:** ^1^ Human Cancer Genomic Research, King Faisal Specialist Hospital and Research Center, Riyadh, Saudi Arabia; ^2^ Department of Pathology, King Faisal Specialist Hospital and Research Centre, Riyadh, Saudi Arabia; ^3^ Department of Oncology Centre, King Faisal Specialist Hospital and Research Centre, Riyadh, Saudi Arabia

**Keywords:** breast cancer, FoxM1, thiostrepton, invasion, apoptosis

## Abstract

Breast cancer (BC) is the most common cause of cancer-related death in females in Saudi Arabia. BC in Saudi women tend to behave more aggressively than breast cancer in the West. Therefore, identification of new molecular targets and treatment strategies are highly warranted to improve patient outcome. FoxM1 has been shown to play a critical role in pathogenesis of various malignancies. In this study, we explored the prevalence and clinical implication of FoxM1 overexpression in Saudi breast cancer. FoxM1 protein overexpression was seen in 79% (770/975) of BC tissues and was associated with aggressive clinical parameters such as younger age (< 30 yrs) (*p* = 0.0172), high grade (*p* < 0.0001), mucinous histology (*p* < 0.0001) and triple negative phenotype (*p* < 0.0001). Overexpression of FoxM1 was significantly associated with activated AKT (*p* < 0.0001), Ki67 expression (*p* < 0.0001), VEGF (*p* < 0.0001), MMP-9 (*p* < 0.0001), XIAP (*p* < 0.0001) and Bcl-xL (*p* = 0.0300). Importantly, FoxM1 overexpression is found to be an independent prognostic marker in multivariate analysis in advanced stage (Stage III and IV) breast cancer (*p* = 0.0298). *In vitro* data using BC cell lines showed that down-regulation of FoxM1 using specific inhibitor, thiostrepton or siRNA inhibited cell migration, invasion and angiogenesis. In addition, treatment of BC cell lines with thiostrepton resulted in inhibition of proliferation and induction of apoptosis in a dose-dependent manner. *In vivo*, thiostrepton treatment regressed MDA-MB-231 cells generated xenografts via down-regulation of FoxM1 and its downstream targets. Our results suggest that FoxM1 may be a potential therapeutic target for the treatment of aggressive breast cancers.

## INTRODUCTION

Breast cancer (BC) is the most common malignancy among females and second leading cause of cancer deaths worldwide [1]. Generally, incidence of BC increases with age and approximately 40% of all patients with BC experience a relapse, of which 10–20% are local and 60–70% are distant metastases [2, 3]. Unlike Western population, BC is more aggressive in Saudi Arabia, presenting at an early age and more advanced stage with higher morbidity and mortality [4, 5]. The poor outcomes in breast cancer are mainly due to the lack of successful therapeutic options following development of resistance to standard therapies (ie, chemotherapy and radiotherapy) and metastatic disease [6]. Identification of new targeted therapy that allows progress in the management of aggressive breast cancer and improves survival is warranted.

Forkhead box M1 (FoxM1) is a transcription factor from FOX protein family which plays an important role in cell proliferation, cell cycle progression, invasion, and metastasis [7–16]. Previous studies demonstrated that elevated FoxM1 expression is found in a variety of cancers, including breast, ovarian, colon, liver, pancreatic, gastric and other cancers [14, 15, 17–21]. Moreover, overexpression of FoxM1 is correlated with a clinically aggressive, drug-resistant, cancer phenotype with poor prognosis [22–25]. However, the clinical relevance of FoxM1 expression has not been investigated in breast cancer in Saudi Arabia, a country with a particularly more aggressive breast cancer and an unusually young age of onset.

The aim of this study is to determine the expression level of FoxM1 in Middle Eastern breast cancer and to examine its association with clinico-pathologic variables as well as to assess its utility as a prognostic indicator. In this study, we have first investigated in detail the expression of FoxM1 in a large cohort of more than 1000 Middle Eastern BC samples using tissue microarray and correlated the data with various clinical and molecular parameters. Our findings indeed confirm the clinical significance of FoxM1 in aggressive Middle Eastern breast cancer. *In vitro* studies were performed using either thiostrepton, a specific FoxM1 inhibitor with proteasomal inhibition activity or siRNA specifically targeting FoxM1 transcript on BC cell lines, to identify FoxM1 as a potential therapeutic target. We demonstrated that down regulation of FoxM1 inhibited cell proliferation, migration, invasion and angiogenesis of BC cell lines. Finally, we have correlated our *in vitro* findings by generating BC cell-xenograft on nude mice and targeted them with thiostrepton. The results presented here help to identify significant role of FoxM1 overexpression in advanced Middle Eastern BC and their use as prognostic marker and therapeutic target in BC.

## RESULTS

### Expression of FoxM1 by immunohistochemistry (IHC) and correlation with clinico-pathological data

**Figure 1:**
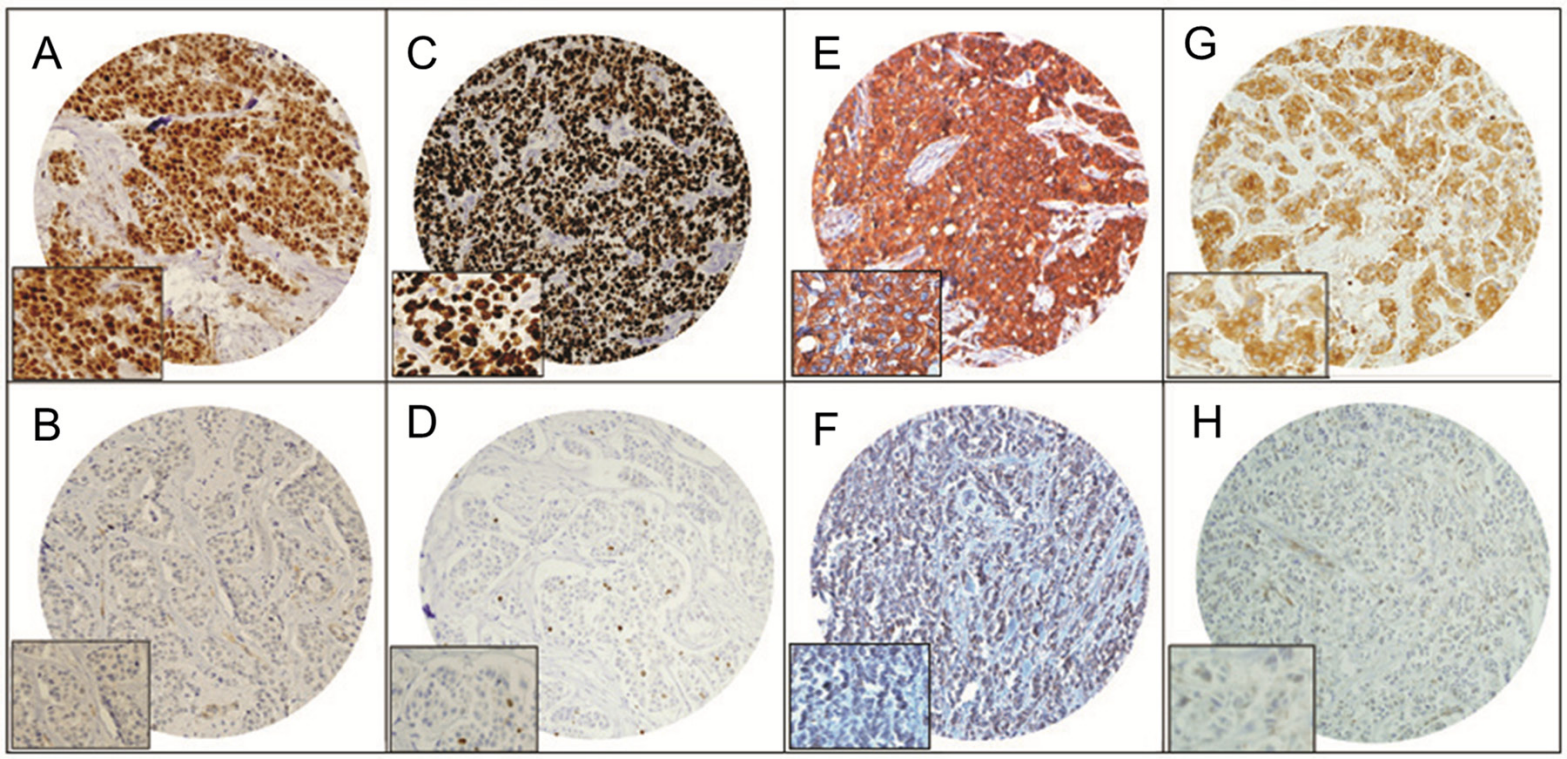
Tissue microarray based immunohistochemistry analysis of FoxM1, Ki-67, VEGF and MMP-9 in breast cancer (BC) patients BC TMA spots showing overexpression of FoxM1 (**A**), Ki-67 (**C**), VEGF (**E**), and MMP-9 (**G**). In contrast, another set of TMA spots showing reduced expression of FoxM1 (**B**), Ki-67 (**D**), VEGF (**F**), and MMP-9 (**H**). 20 X/0.70 objective on an Olympus BX 51 microscope. (Olympus America Inc, Center Valley, PA, USA) with the inset showing a 40X 0.85 aperture magnified view of the same TMA spot.

We first sought to determine over-expression of FoxM1 in a cohort of clinical BC samples by IHC in a tissue microarray format. Using a TMA of 1009 samples, FoxM1 staining was interpretable in 975 spots and FoxM1 was found to be over-expressed in 79% (770/975) of cases and was found to be significantly associated with younger age (< 30 years) (*p* = 0.0172), poorly differentiated BC (*p* < 0.0001), mucinous histology (*p* < 0.0001) and TNBC (p < 0.0001), however, there was no association with tumor size, nodal involvement and metastasis (Table 1). At the molecular level, FoxM1 over-expression was significantly associated with XIAP (*p* < 0.0001), p-AKT (*p* = 0.0001), Bcl-xL (*p* = 0.0300), VEGF (*p* < 0.0001), MMP-9 (*p* < 0.0001) and proliferative marker, Ki67 (*p* < 0.0001) over-expression (Table 1 and Figure 1). BC patients showing FoxM1 over-expression showed poor overall survival compared to cases not expressing this protein but this difference did not reach statistical significance (*p* = 0.1044) (Table 1). However, when we examined late stage (Stage III and IV) BC cases in our cohort of samples, our data showed that FoxM1 over-expression was 76.8% and significantly associated with younger age (*p* = 0.0033), poorly differentiated tumors (*p* < 0.0001), mucinous histology (*p* = 0.0003) and TNBC (*p* = 0.0008) as well as a poor overall 5 year survival (*p* = 0.0033) (Table 2 and Supplementary Figure 1). On multivariate analysis using the Cox proportional hazards model, FoxM1 overexpressing cases in late stage demonstrated significant poor survival when adjusted for age, histology, tumor grade and TNBC (hazard ratio, 1.82; 95% confidence interval [95% CI], 1.06-3.37 [*p* = 0.0298]) (Supplementary Table 2).

**Table 1:** Correlation of FoxM1 with clinico-pathological parameters in breast cancer

	Total		High		Low		*P* value
	*N*	%	*N*	%	*N*	%	
**Total Number of Cases**	975		770	79.0	205	21.0	
**Age Groups**							
< 30	54	5.5	49	90.7	5	9.3	0.0172
30	921	94.5	721	78.3	200	21.7	
**Tumor size**							
T1	207	21.7	168	81.2	39	18.8	0.6978
T2	481	50.5	380	79.0	101	21.0	
T3	141	14.8	107	75.9	34	24.1	
T4	124	13.0	97	78.2	27	21.8	
**Lymph Nodes**							
N0	302	33.1	243	80.5	59	19.5	0.2017
N1	295	32.3	235	79.7	60	20.3	
N2	189	20.7	138	73.0	51	27.0	
N3	127	13.9	103	81.1	24	18.9	
**Metastasis**							
M0	785	89.8	620	79.0	165	21.0	0.5778
M1	89	10.2	68	76.4	21	23.6	
**Tumour Stage**							
I	75	8.8	64	85.3	11	14.7	0.3992
II	371	43.8	292	78.7	79	21.3	
III	312	36.8	240	76.9	72	23.1	
IV	89	10.5	68	76.4	21	23.6	
**Recurrence**							
Yes	253	29.5	216	85.4	37	14.6	0.0028
No	605	70.5	463	76.5	142	23.5	
**Extra Nodal Ext.**							
Yes	266	33.2	210	79.0	56	21.0	0.9438
No	536	66.8	422	78.7	114	21.3	
**LVI**							
Yes	359	41.4	280	78.0	79	22.0	0.8587
No	507	58.6	398	78.5	109	21.5	
**Histological Grade**							
Well differentiated	76	7.9	64	85.3	11	14.7	< 0.0001
Moderately differentiated	495	51.3	370	74.7	125	25.3	
Poorly differentiated	393	40.8	355	90.3	38	9.7	
**Histology**							
Infiltrating Ductal Carcinoma	888	93.8	712	80.2	176	19.8	< 0.0001
Infiltrating Lobular	43	4.5	22	51.2	21	48.8	
Mucinous Ca	16	1.7	15	93.8	1	6.2	
**Triple Negative**							
No	826	85.1	633	76.6	193	23.4	< 0.0001
Yes	145	14.9	135	93.1	10	6.9	
**Ki-67**							
Above 30	619	64.7	555	89.7	64	10.3	< 0.0001
Below = 30	337	35.3	205	60.8	132	39.2	
**XIAP**							
Above 85	283	29.8	256	90.5	27	9.5	< 0.0001
Below = 85	667	70.2	503	75.4	164	24.6	
**phos_AKT (473)**							
Negative	729	77.5	558	76.5	171	23.5	0.0001
Positive	212	22.5	187	88.2	25	11.8	
**MMP-9**							
Above 50	670	72.0	584	87.2	86	12.8	< 0.0001
Below = 50	261	28.0	151	57.8	110	42.2	
**VEGFA**							
Above 100	709	78.7	589	83.1	120	16.9	< 0.0001
Below = 100	192	21.3	132	68.7	60	31.3	
**BCL-Xl**							
Above 220	268	28.97	228	85.07	40	14.93	0.0300
Below = 220	657	71.03	519	79.00	138	21.00	
**Survival**							
OS 5 Years				78.2		86.5	0.1044

**Table 2:** Correlation of FoxM1 with clinico-pathological parameters in late stage breast cancer (Stage III+IV)

	Total		High		Low		*P* value
	*N*	%	*N*	%	*N*	%	
**Total Number of Cases**	401		308	76.8	93	23.2	
**Age Groups**							
< 30	16	4.0	16	100.0	0	0.0	0.0033
30	385	96.0	292	75.8	93	24.2	
**Tumor size**							
T1	39	9.8	29	74.4	10	25.6	0.9715
T2	151	37.7	117	77.5	34	22.5	
T3	106	26.5	81	76.4	25	23.6	
T4	104	26.0	81	77.9	23	22.1	
**Lymph Nodes**							
N0	23	6.1	20	87.0	3	13.0	0.1827
N1	81	21.5	63	77.8	18	22.2	
N2	168	44.4	122	72.6	46	27.4	
N3	106	28.0	87	82.1	19	17.9	
**Metastasis**							
M0	312	77.8	240	76.9	72	23.1	0.9187
M1	89	22.2	68	76.4	21	23.6	
**Tumour Stage**							
III	312	77.8	240	76.9	72	23.1	0.9187
IV	89	22.2	68	76.4	21	23.6	
**Extra Nodal Ext.**							
Yes	190	50.8	152	80.0	38	20.0	0.2007
No	184	49.2	137	74.5	47	25.5	
**LVI**							
Yes	213	57.9	170	79.8	43	20.2	0.2045
No	155	42.1	115	74.2	40	25.8	
**Recurrence**							
Yes	144	39.4	128	88.9	16	11.1	< 0.0001
No	221	60.6	155	70.1	66	29.9	
**Histological Grade**							
Well differentiated	21	5.3	8	38.1	13	61.9	< 0.0001
Moderately differentiated	205	51.5	145	70.7	60	29.3	
Poorly differentiated	172	43.2	154	89.5	18	10.5	
**Histology**							
Infiltrating Ductal Carcinoma	372	94.2	294	79.0	78	21.0	0.0003
Infiltrating Lobular	21	5.3	8	38.1	13	61.9	
Mucinous Ca	2	0.5	2	100.0	0	0	
**Triple Negative**							
No	336	84.4	249	74.1	87	25.9	0.0008
Yes	62	15.6	57	91.9	5	8.1	
**Ki-67 IHC**							
Above 30	261	66.6	236	90.4	25	9.6	< 0.0001
Below = 30	131	33.4	68	51.9	63	48.1	
**XIAP**							
Above 85	123	31.5	110	89.4	13	10.6	< 0.0001
Below = 85	268	68.5	192	71.6	76	28.4	
**phos_AKT (473)**							
Negative	278	72.2	203	73.0	75	27.0	0.0005
Positive	107	27.8	95	88.8	12	11.2	
**MMP-9**							
Above 50	266	69.8	228	85.7	38	14.3	< 0.0001
Below = 50	115	30.2	64	55.6	51	44.4	
**VEGFA**							
Above 100	279	74.0	229	82.1	50	17.9	0.0017
Below = 100	98	26.0	65	66.3	33	33.7	
**Survival**							
OS 5 Years				60.2		83.0	0.0033

### Inhibition of FoxM1 in BC cells decreased invasion, migration and angiogenesis

**Figure 2:**
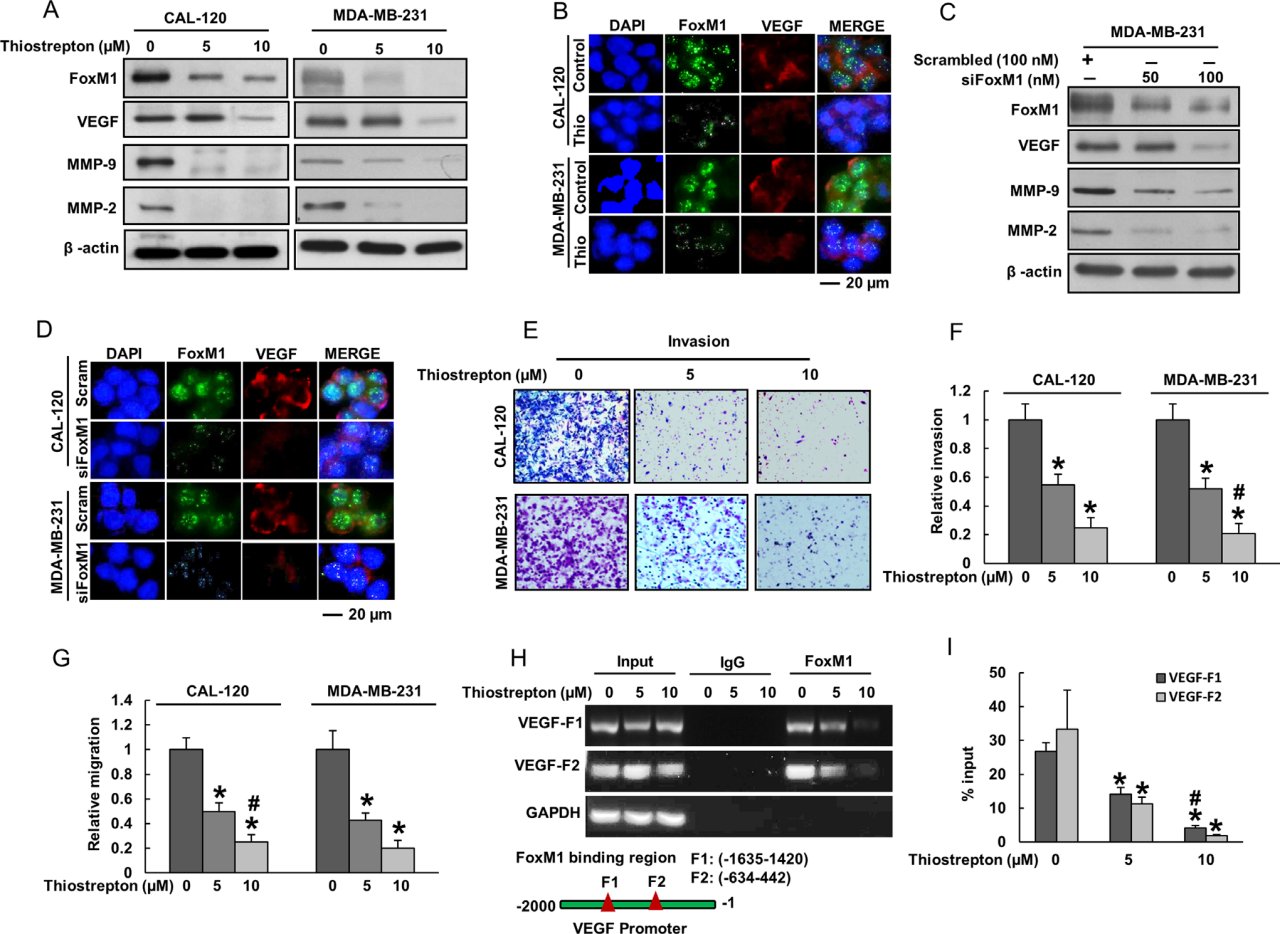
Inhibition of FoxM1 in BC cells decreased invasion, migration and angiogenesis (**A**) Thiostrepton inhibits the expression of FoxM1, VEGF, MMP-9 and MMP-2 in BC cells. CAL-120 and MDA-MB-231 cells were treated with 5 and 10 mM thiostrepton for 48 hours. After cell lysis, equal amounts of proteins were separated on SDS-PAGE, and immunoblotted with antibodies against FoxM1, VEGF, MMP-9, MMP-2 and Beta-actin as indicated. (**B**) Representative images of fluorescence immunostaining for FoxM1 and VEGF in BC cells after treatment with thiostrepton. (**C**) FoxM1 siRNA transfection down-regulated expression of FoxM1, VEGF, MMP-9 and MMP-2 in BC cells. MDA-MB-231 cells were transfected with scrambled siRNA (100 nM) and FoxM1 siRNA (50 nM and 100 nM) with Lipofectamine 2000. After transfection, cells were lysed and equal amounts of proteins were separated by SDS-PAGE, and immunoblotted with antibodies against FoxM1, VEGF, MMP-9, MMP-2 and Beta-actin as indicated. (**D**) Representative images of fluorescence immunostaining for FoxM1 and VEGF in BC cells after post-transfection with FoxM1 siRNA. (**E**–**G**) Invasion-migration assays were performed using CAL-120 and MDA-MB-231 cells treated with 5 and 10 µM thiostrepton for 48 hours as described in Materials and Methods. (**H**–**I**) FoxM1 binding to VEGF promoter. For the ChIP assay, the FoxM1 binding regions on VEGF promoter were identified. MDA-MB-231 cells were treated with and without thiostrepton (5 and 10 μm) for 24 h, fixed with formaldehyde, and cross-linked, and then chromatin was isolated. The chromatin was immunoprecipitated (IP) with an anti-FoxM1 antibody or control mouse IgG. The FoxM1 binding to the VEGF promoters was analyzed by regular PCR (H) or quantitative real time PCR (I) with a primer specific for the FoxM1 binding regions in VEGF promoter. The data represent the percent input and are normalized to each control. GAPDH was used as a loading control. Data presented in bar graphs are the mean ± SD of three independent experiments. ^*^ and ^#^ indicate statistically significant differences compared to control without treatment or thiostrepton (5 µM) treatment, respectively with *p* < 0.05.

It is known that in addition of increasing proliferation of cancer cells [26–28], FoxM1 plays a major role in increasing the capability of cancer cells to invade surrounding tissues and migrate to other regions thereby increasing the metastatic potential in various cancers [28–31]. Therefore, targeting FoxM1 expression can lead to inhibition of these invasion and migration properties of cancer cells. Our clinico-pathological data showed that FoxM1 over-expression was significantly associated with VEGF and MMP-9 expression, proteins implicated in tumor angiogenesis and extracellular matrix degradation respectively, thereby allowing cancer cells to invade and migrate to surrounding tissue [32, 33]. Therefore, we sought to determine whether FoxM1 down-regulation using thiostrepton, a specific inhibitor of FoxM1 with additional proteasomal activity [34] affects the expression of VEGF, MMP-9 and MMP-2 that plays an important role in invasion, migration and angiogenesis. In our study, treatment of thiostrepton markedly down-regulated the expressions of FoxM1, VEGF, MMP-9 and MMP-2 in both BC cells, CAL-120 and MDA-MB-231 (Figure 2A). Similar results were observed by immunofluorescence where down-regulation of FoxM1 using thiostrepton decreases VEGF expression in BC cells (Figure 2B). This data was further confirmed by using specific FoxM1 siRNA knockdown in BC cells showing similar results (Figure 2C and 2D).

To investigate whether down regulation of FoxM1 plays a role in inhibiting invasion and migration, BC cell lines were treated with different doses of thiostrepton. Interestingly, inhibition of FoxM1 using thiostrepton significantly decreased invasion (Figure 2E and 2F) and migration (Figure 2G) of BC cells. These data suggest that thiostrepton treatment of BC cells decreases the ability of cancer cells to spread to local and surrounding areas due to down-regulation of FoxM1. To determine whether FoxM1 transcriptionally activates VEGF in our model, we performed ChIP analysis. Previous reports indicated the presence of two putative FoxM1 binding regions in the VEGF promoter [33]. As shown in Figure 2H, ChIP analysis demonstrated that FoxM1 binds to VEGF promoters at both sites, F1 (-1635-1420) and F2 (-634-442) in BC cells. Interestingly, the degree of FoxM1 binding to VEGF promoter at both sites was decreased after thiostrepton treatment in a dose dependent manner (Figure 2H and 2I).

### Inhibition of FoxM1 in BC cells decreased HUVECs chemotactic migration, invasion and tube formation

Our data showed that FoxM1 over-expression was significantly associated with VEGF in BC patient samples and inhibition of FoxM1 drastically downregulated VEGF expression in BC cells. VEGF plays an important role during neo-angiogenesis through its mitogenic effect on endothelial cells [35]. Therefore, we sought to investigate whether down-regulation of FoxM1 in BC cells could inhibit chemotactic migration, invasion, and tube formation of HUVECs. For this, BC cell lines were treated with thiostrepton or FoxM1 siRNA for indicated doses. After treatment, conditioned media was collected and used for HUVECs chemotactic migration, invasion, and tube formation experiments.

**Figure 3:**
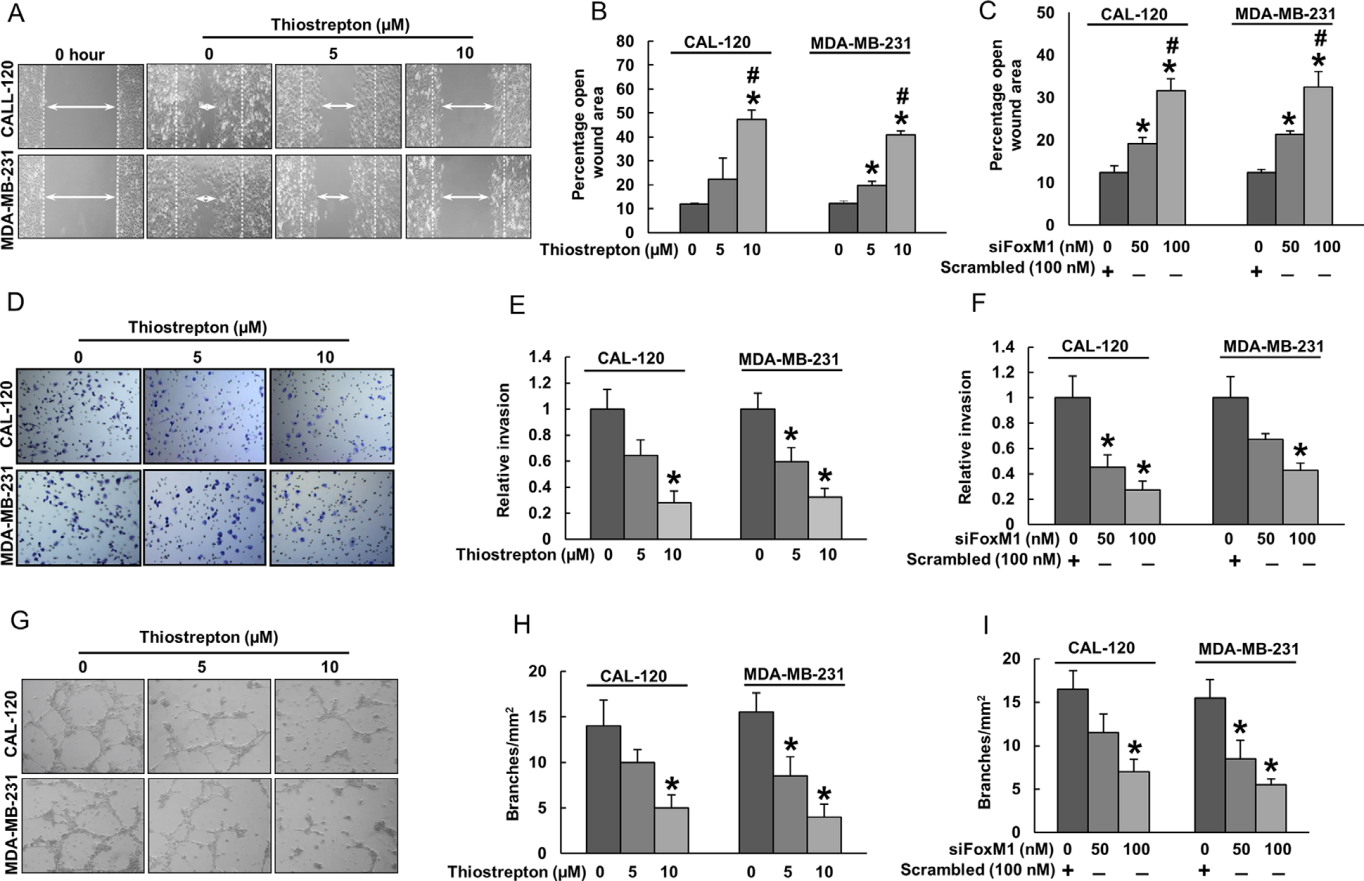
Inhibition of FoxM1 in BC cells decreased HUVECs chemotactic migration, invasion, and tube formation (**A**–**C**) Inhibition of FoxM1 decreased HUVECs migration. HUVECs were grown into wells of collagen coated 24 well plate dishes to 100% confluence. Cells were starved to inactivate cell proliferation and then wounded by 200 µl pipette tips. Cells were treated with conditioned media from BC cells treated with thiostrepton or FoxM1 siRNA. Images were taken at 0 h and 24 h following wounding. The wound area was measured using Image J software, and data was expressed as percentage of open wound area calculated by dividing migrated distance by scratched distance. (**D**–**F**) Inhibition of FoxM1 decreased HUVECs invasion. HUVECs (10^5^ cells/Transwell) were seeded into the upper compartment of invasion. The bottom chambers were filled with conditioned media from BC cells treated with thiostrepton or FoxM1 siRNA. After 24 h incubation, migrated cells were fixed, stained and quantified. (**G**–**I**) FoxM1 inhibition caused inhibition of HUVECs tube formation. HUVECs grown on matrigel were treated with conditioned media from thiostrepton or FoxM1 siRNA treated BC cells for 24 h, cells were fixed, and tubular structures were photographed and quantified. Data presented in bar graphs are the mean ± SD of three independent experiments. ^*^ and ^#^ indicate statistically significant differences compared to control/scramble siRNA without treatment or thiostrepton (5 µM)/siFoxM1 (50 nM) treatment, respectively with *p* < 0.05.

Effect of FoxM1 downregulation on the chemotactic motility of HUVECs is shown in Figure 3A–3C. HUVECs migrated into the clear area when stimulated with conditioned media (source of VEGF) of BC cells whereas, less migration was observed in wells treated with conditioned media from thiostrepton (Figure 3A and 3B) or FoxM1 siRNA treated (Figure 3C) BC cells. HUVECs showed a high invasive property through the collagen matrix when treated with conditioned media from BC cells (Figure 3D–3F). Treatment with conditioned media from thiostrepton (Figure 3D and 3E) or FoxM1 siRNA treated (Figure 3F) BC cells caused less invasion of HUVECs in a dose dependent manner.

The tubular formation of endothelial cells is also a key step of angiogenesis [35]. Incubation of HUVECs with conditioned media (source of VEGF) of BC cells resulted in the formation of elongated and tube like structures which were effectively reduced by conditioned media from thiostrepton (Figure 3G and 3H) or FoxM1 siRNA (Figure 3I) treated BC cells. To further confirm these observations, we determined the secreted levels of VEGF by ELISA experiment in thiostrepton or FoxM1 siRNA treated BC cells (Supplementary Figure 2A–2B). Decreased levels of VEGF was observed in a dose dependent manner in thiostrepton (Supplementary Figure 2A) or FoxM1 siRNA (Supplementary Figure 2B) treated BC cells. Together, these findings suggest that inhibition of FoxM1 can significantly suppresses endothelial cell invasion, migration and angiogenesis by down-regulating VEGF.

### Thiostrepton treatment of BC cells caused inhibition of cell viability and induction of caspase-dependent apoptosis

**Figure 4:**
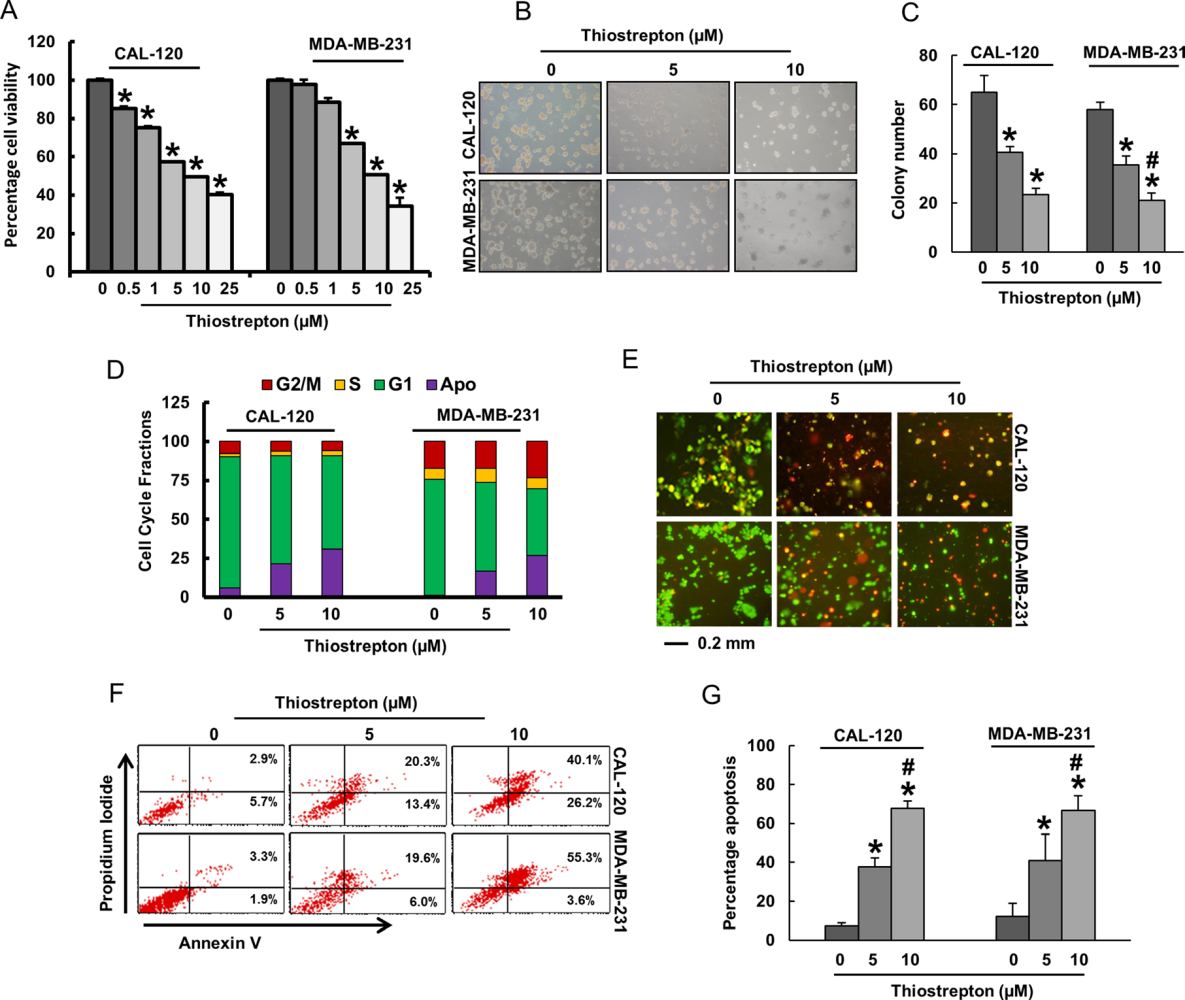
Thiostrepton treatment inhibited cell viability via induction of apoptosis in BC cells (**A**) Thiostrepton induced inhibition of cell viability in BC cells. CAL-120 and MDA-MB-231 cells were incubated with different doses of thiostrepton (0.5–25 μM). Cell viability assays were performed using MTT as described in Materials and methods. The graph displays the mean +/- SD (standard deviation) of three independent experiments with replicates of six wells for all the doses and vehicle control for each experiment ^*^p < 0.01, statistically significant (Students *t*-test). (**B**–**C**) Effect of thiostrepton on clonogenicity of BC cells. BC cells were treated with 5 and 10 μM thiostrepton and plated in soft agar for 4 weeks. After incubation, clonogenic assays were performed using as described in Materials and Methods. (**D**) Cell cycle analysis of BC cells treated with thiostrepton. CAL-120 and MDA-MB-231 cells were treated with 5 and 10 μM thiostrepton for 48 hours. Following incubation, cells were analysed for cell cycle fractions by flow cytometry. (**E**) CAL-120 and MDA-MB-231 cells were treated with 5 and 10 mM thiostrepton for 48 hours. Following treatment, cells were stained with Calcein AM and Ethidium homodimer as described in Material and Methods section and cells were analysed under a fluorescent microscope with a long-pass filter. (**F**–**G**) Thiostrepton–mediated apoptosis in BC cells. CAL-120 and MDA-MB-231 cells were treated with 5 and 10 µm thiostrepton for 48 hours and cells were subsequently stained with fluorescein-conjugated annexin-V and propidium iodide and analysed by flow cytometry. Data presented in bar graphs are the mean ± SD of three independent experiments. ^*^ and ^#^ indicate statistically significant differences compared to control without treatment or thiostrepton (5 µM) treatment, respectively with *p* < 0.05.

After analyzing the clinical data, we sought to determine whether targeting FoxM1 expression in BC cells can be used as a viable therapeutic strategy to inhibit cell viability and induce apoptosis in addition to inhibition of invasion and migration. We therefore treated BC cell lines, CAL-120 and MDA-MB-231, with increasing doses of thiostrepton for 48 hours and investigated cell viability by MTT assay. As shown in Figure 4A, there was a significant (*p* < 0.05) dose-dependent inhibition of cell viability in both the cell lines. Using doses of 5 and 10 μM thiostrepton and 48 hours’ time point, the rest of the experiments were performed. We next examined colony formation in BC cells following treatment with 5 and 10 μM thiostrepton. Following treatment with thiostrepton, cells were plated in agarose gel and allowed to grow at 37^o^ C for 4 weeks following which cells were stained and visualized on an inverted microscope. BC cells treated with thiostrepton lost their ability to form colonies in both the cell lines (Figure 4B and 4C).

We wanted to determine whether inhibition of cell viability was due to cell cycle arrest or apoptosis. For this reason, we treated both BC cells with 5 and 10 μM thiostrepton for 48 hours and analyzed the cells for cell cycle by flow cytometry. Our data showed that there was a decrease in the G1 and G2 fraction with concurrent shift of cells in Sub-G1 fraction depicting apoptosis [36] (Figure 4D). We also analyzed cell death by Live Dead assay following treatment with thiostrepton to confirm that cells following treatment were actually dying and the decreased cell counts were not due to cell inhibition (Figure 4E). Finally, to confirm apoptosis in thiostrepton treated BC cells, we stained CAL-120 and MDA-MB-231 cells with annexin V and Propidium iodide following treatment with 5 and 10 μM thiostrepton for 48 hours and analyzed by flow cytometry. As shown in Figure 4F–4G, there was an increase in apoptotic cells following treatment with thiostrepton in both the cell lines confirming that these cell death was due to apoptosis.

**Figure 5:**
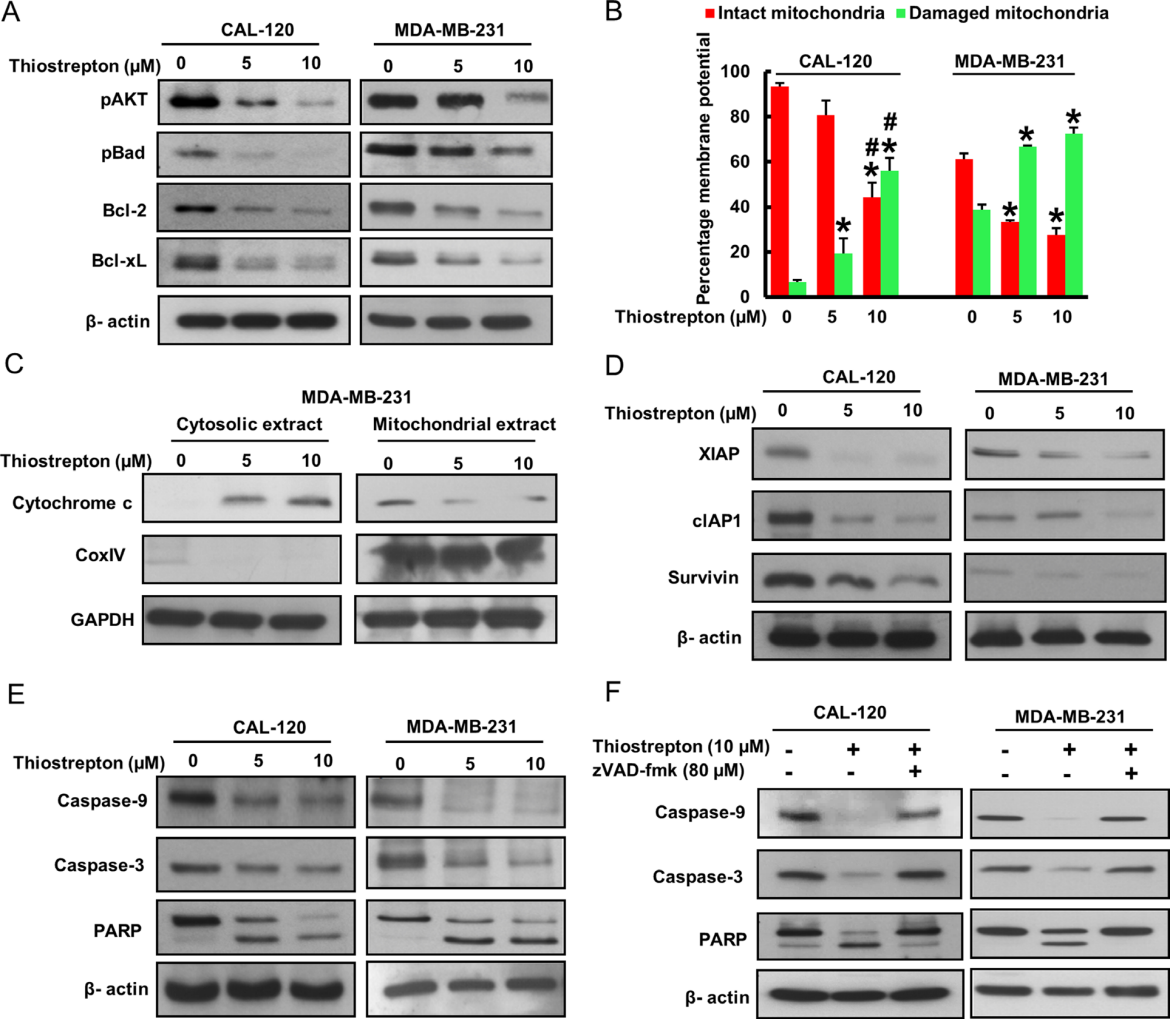
Thiostrepton treatment induces caspase-dependent apoptosis via activation of mitochondrial apoptotic pathway (**A**) FoxM1 down-regulation leads to inactivation of AKT and down-stream target Bad in BC cells. CAL-120 and MDA-MB-231 cells were treated with 5 and 10 μM thiostrepton for 48 hours. Following treatment, cells were lysed and immunoblotted with antibodies against p-AKT, p-Bad, Bcl-2, Bcl-xL and Beta-actin. (**B**) Estimation of mitochondrial membrane potential by flow cytometry. BC cells were treated with 5 and 10 μM thiostrepton for 48 hours. Following treatment, cells were stained with 10 μM JC1 and analysed by flow cytometry. (**C**) Thiostrepton-induced release of cytochrome c. MDA-MB-231 cells was treated with and without 5 and 10 mM thiostrepton for 48 hours. Mitochondrial free, cytosolic fractions and mitochondrial extracts were isolated as described in Materials and Methods. Cell extracts were separated on SDS-PAGE, transferred to PVDF membrane, and immunoblotted with an antibody against cytochrome c, Cox IV and GAPDH. (**D**) BC cells were treated with thiostrepton (5 and 10 μM) for 48 hours and expression of XIAP, cIAP1 and Survivin were determined by immunoblotting. Beta-actin was used for equal loading. (**E**) Activation of caspases and cleavage of PARP induced by thiostrepton treatment in BC cells. BC cells were treated with and without 5 and 10 mM thiostrepton for 48 hours. Cells were lysed, equal amount of proteins were separated on SDS-PAGE and immuno-blotted with antibodies against caspase-9, pro-caspase-3, PARP, and Beta-actin. Each experiment was repeated three times to confirm reproducibility. (**F**) BC cells were pre-treated with either 80 mM z-VAD/fmk for 2 hours and treated with 10 mM of thiostrepton for 48 hours and cells were lysed, equal amount of proteins were separated on SDS-PAGE and immuno-blotted with antibodies against pro-caspase-3, PARP and Beta-actin. Data presented in bar graphs are the mean ± SD of three independent experiments. ^*^ and ^#^ indicate statistically significant differences compared to control without treatment or thiostrepton (5 µM) treatment, respectively with *p* < 0.05.

Once the data generated confirmed that BC cells treated with thiostrepton were actually undergoing apoptosis, we were interested in identifying the apoptotic pathway that was being activated by thiostrepton in inducing apoptosis. Our clinico-pathological data showed a significant association between FoxM1 over-expression and activated AKT. It has been previously shown that phosphorylation of AKT inhibits apoptosis via activation of Bad [37]. We therefore examined the activation status of AKT and Bad in BC cells following treatment with 5 and 10 μM thiostrepton for 48 hours. As shown in Figure 5A, there was inactivation of AKT and Bad in thiostrepton treated BC cells as determined by Western blotting. Once Bad protein is inactivated, the apoptotic signal reaches the mitochondria and causes down-regulation of anti-apoptotic proteins, Bcl-2 and Bcl-xL. Our data showed that there was down-regulation of expression of Bcl-2 and Bcl-xL following treatment with thiostrepton (Figure 5A). After the apoptotic signal reaches the mitochondria, it elicits its disruptive function leading to change in the mitochondrial membrane potential leading to damage. Therefore, we investigated the changes in membrane potential of the mitochondria following treatment with thiostrepton. We found there was increased mitochondrial damage after thiostrepton treatment in both cell lines studied as depicted by an increase in green stained cells (apoptotic cells) as compared to red stained cells (normal cancer cells) (Figure 5B). This data confirmed that the mitochondrion was indeed getting damaged following down-regulation of anti-apoptotic proteins, Bcl-2 and Bcl-xL, as a consequence of FoxM1 down-regulation. Once the mitochondrion is damaged, there is release of cytochrome c into cytosol thereby leading to activation and cleavage of caspase-9. To ascertain these findings, we treated BC cells with 5 and 10 μM thiostrepton for 48 hours and isolated cytosolic as well as mitochondrial extracts. As shown in Figure 5C, there was decrease in expression of cytochrome c in mitochondrial extracts following treatment with thiostrepton with concurrent increase of cytochrome c in the cytosolic compartment confirming mitochondrial damage and cytochrome c release. In order for the intrinsic apoptotic pathway to be efficiently activated, Inhibitor of Apoptosis Proteins (IAPs) also needs to be down regulated following treatment with thiostrepton. Therefore, we examined expression of IAPs; XIAP, cIAP1 and survivin in BC cells following treatment with 5 and 10 μM thiostrepton for 48 hours. There was down-regulation of the IAPs in both cell lines examined (Figure 5D).

Once cytochrome c is released into cytosol and down-regulation of IAPs, there is activation and cleavage of down-stream caspases and PARP. To confirm this, total cell lysate of CAL-120 and MDA-MB-231 cells following treatment with 5 and 10 μM thiostrepton were immunoblotted with antibodies against caspases-9, -3 and PARP. There was a cleavage of caspases-9, -3 and PARP in both the cell lines treated with thiostrepton confirming caspase-dependent apoptosis in these cells (Figure 5E). To further confirm caspase dependent apoptosis in thiostrepton treated BC cells, we pre-treated BC cells with a universal inhibitor of caspase activation, zVAD-fmk, for three hours followed by treatment with 10 μM thiostrepton for 48 hours. As compared to thiostrepton treated cells, there was inhibition of caspase activation and apoptosis in cells pretreated with zVAD-fmk confirming the role of caspases in thiostrepton-induced apoptosis in BC cells (Figure 5F).

### Down-regulation of FoxM1 regress *in vivo* BC xenografts

**Figure 6:**
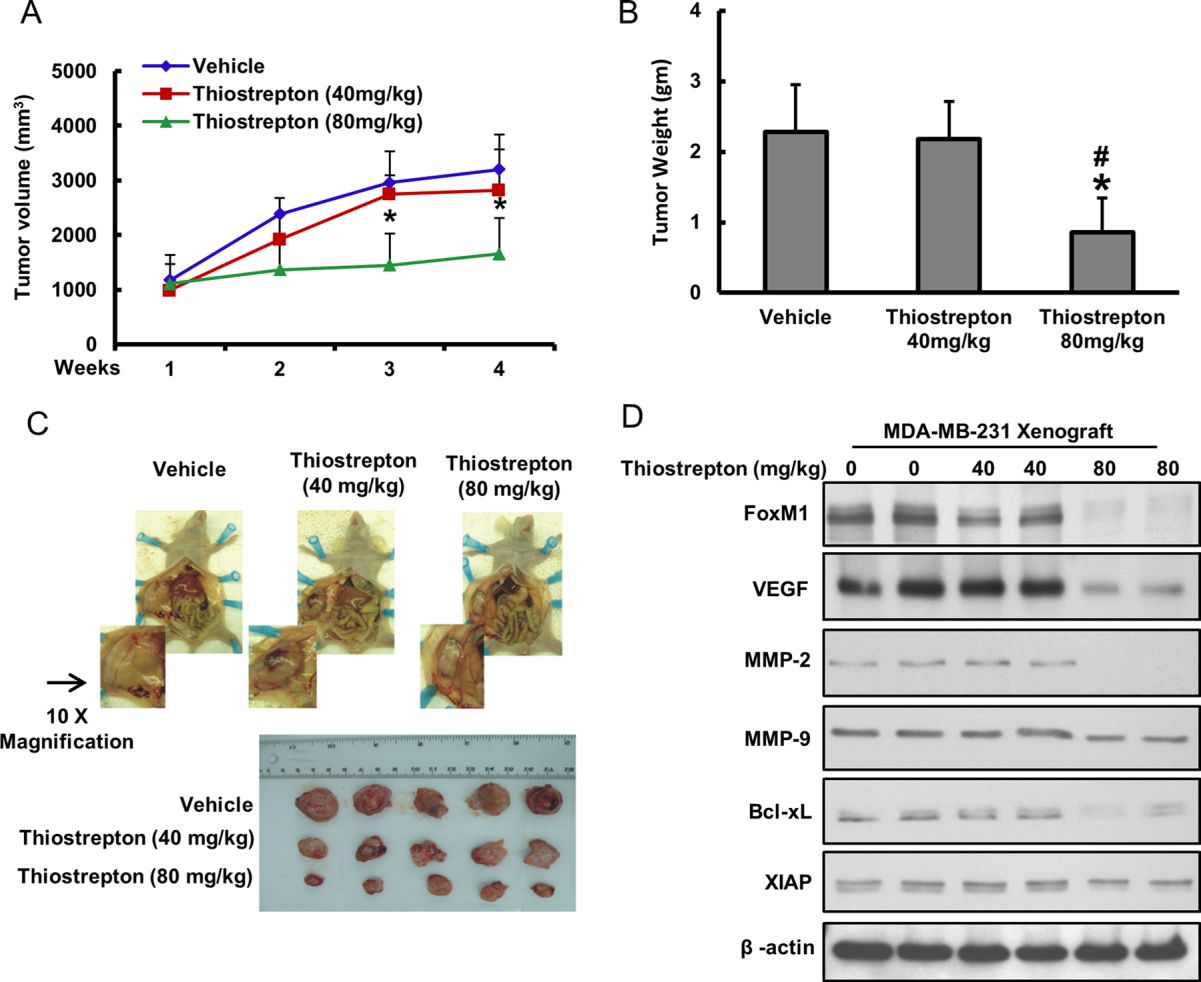
Thiostrepton-inhibits growth of MDA-MB-231 Xenografts Female nude mice at 6 weeks of age were injected subcutaneuosly with 10 × 10^6^ MDA-MB-231 cells. After one week, mice were treated with thiostrepton 40 mg/kg/dose, 80 mg/kg/dose and with 5% DMSO in PBS as a vehicle control, (**A**) Volume of each tumor was measured every week. The average (*n* = 5) tumor volume of mice was calculated, ^*^p < 0.05 inhibition of BC tumor growth by thiostrepton. (**B**) After 4 weeks of treatment, mice were sacrificed and tumor weights were measured, ^*^p < 0.05 compared to vehicle-treated mice by Student’s *t*-test. (**C**) Representative tumor images of vehicle and thiostrepton treated mice. (**D**) Whole cell lysate from mice treated with vehicle, thiostrepton (40 mg/kg) and thiostrepton (80 mg/kg) were isolated and 10 µg protein were separated by SDS-PAGE, transferred to PVDF membrane, and immunoblotted with antibodies against FoxM1, VEGF, MMP-2, MMP-9, Bcl-xL, XIAP and Beta-actin. Data presented in bar graphs are the mean ± SD of two independent experiments. ^*^ and ^#^indicate statistically significant differences compared to vehicle or thiostrepton (40 mg/kg) treatment, respectively with *p* < 0.05.

Our *in vitro* data clearly indicated an important role of FoxM1 downregulation by thiostrepton in inhibiting the invasion/migration capability and inducing apoptosis in BC cells. To further confirm these findings *in vivo*, we conducted experiments using nude female mice by generating xenografts of BC cell line, MDA-MB-231, and treating them with two different doses of thiostrepton for a period of 4 weeks. After 4 weeks, the animals were sacrificed and their tumor weight was measured and proteins isolated. None of the animals experienced any weight loss, decreased activity or loss of appetite. We found that thiostrepton treatment decreased tumor volume (Figure 6A) and weight (Figure 6B) at both doses used; however, doses of 80 mg/kg body weight caused a significant reduction of tumor volume and growth. Naked eye examination of post-necropsy tumors also clearly showed a reduction in size of tumor treated with thiostrepton as compared to vehicle treated tumors (Figure 6C). Finally, proteins isolated from tumors showed down-regulation of FoxM1, VEGF, MMP-2, MMP-9, Bcl-xL, XIAP and β-actin following treatment with thiostrepton indicating that targeting FoxM1 can regress BC xenografts without any toxicity (Figure 6D).

## DISCUSSION

Breast cancer (BC) continues to be the leading cause of morbidity in females in Saudi Arabia [38] and all over the world with an approximation of more than 450,000 death per year [39]. Despite improvement in the treatment of breast cancer, the desired results have still not been achieved thereby prompting identification of new molecular targets that can be therapeutically utilized to improve the overall survival of breast cancer in this region. In order to identify these molecular targets, we analyzed a large cohort of breast cancer cases and found that FoxM1 was over-expressed in 79% of all BC studied. Previous studies have shown the expression and the prognostic significance of FoxM1 in several types of cancer including breast cancer [14, 15, 17–21]. However, the clinico-pathological significance of FoxM1 expression in Middle Eastern population has not been evaluated. Our results indicate that FoxM1 was over-expressed in a very high percentage of BC and was significantly associated with aggressive parameters like younger age, poorly differentiated tumors, mucinous histology and TNBC.

At the molecular level, we found that FoxM1 over-expression was significantly associated with Ki67, XIAP, p-AKT, MMP-9, Bcl-xL and VEGF. These data clearly indicate that FoxM1 over-expression in BC is an indicator of aggressive phenotype that warrants urgent intervention for management. Even though, FoxM1 over-expression was not found to be associated with overall poor survival, however, we found FoxM1 expression to be an independent marker for poor survival in late stage disease (Stage III and IV) in multivariate analysis. These findings also highlight the importance of identifying this subgroup of breast cancer with over-expression of FoxM1 early to adequately manage the disease before the disease progresses into a life threatening cancer.

In our study, we also detected a significant association between FoxM1 and aggressive parameters such as recurrence, poorly differentiated tumors, mucinous histology and TNBC in advanced stage BC (Stage III and IV). In addition, FoxM1 overexpression was also significantly associated with Ki67, XIAP, p-AKT, MMP-9 and VEGF in advanced stage BC. Previous studies have shown the association of FoxM1 with VEGF, MMP-9 and MMP-2 in various cancers leading to increased invasiveness and migratory ability of cancer cells causing metastasis, and targeting FoxM1 has been shown to inhibit this phenomena via down-regulation of VEGF, MMP-9 and MMP-2 [7, 26, 33, 40]. Our data is in concordance with these finding as we found a significant association between FoxM1 overexpression and VEGF and MMP-9 in our cohort of breast cancer cases.

Targeting FoxM1 using thiostrepton, a thiazole antibiotic has been shown to be beneficial in various cancers [41–43]. We also found that targeting FoxM1 expression with thiostrepton inhibited cell viability and induced mitochondrial dependent apoptosis in BC cells at doses that have previously been shown not to induce apoptosis in peripheral blood mononuclear cells [44]. Thiostrepton treatment caused damage to the mitochondrial membrane via conformational changes and upregulation of Bax with subsequent down-regulation of Bcl-2 and Bcl-xL, leading to release of cytochrome c and activation of caspases-9 and -3 and eventually apoptosis. In addition to apoptosis, transcriptional down-regulation of FoxM1 also inhibited invasiveness, migratory and angiogenic capabilities of BC cells independent of apoptosis via down-regulation of VEGF, MMP-9 and MMP-2. Interestingly, VEGF transcriptional activation by FoxM1 is important for BC angiogenesis. These data clearly highlight the utility of thiostrepton, not only as an inducer of apoptosis but also as an agent that can decrease the ability of FoxM1 expressing cells to invade surrounding tissues eventually leading to metastasis.

Our *in vivo* data on BC xenografts treated with thiostrepton not only validated our *in vitro* findings by causing regression of tumor size and volume via down-regulation of FoxM1 and its downstream targets; VEGF, MMP-9, MMP-2, Bcl-xL and XIAP, but also gave an indication that thiostrepton can be used in humans as the doses of thiostrepton used did not induce toxicity, weight loss or lethargy in the experimental animals. These data clearly indicate the importance of targeting FoxM1 using thiostrepton for management of BC in the future.

In summary, our results show that FoxM1 expression was found to be increased in Middle Eastern BC. We also presented experimental evidence that strongly support the role of FoxM1 in tumor progression and metastasis of BC. The current study demonstrates that down regulation of FoxM1 could potentially be an effective approach for inhibiting VEGF, MMP-2 and MMP-9 which is likely to result in inhibition of cell growth, invasion, angiogenesis and metastasis of BC. Targeting FoxM1 over-expressing BC with thiostrepton either accompanied with chemotherapy or as maintenance after chemotherapy maybe beneficial in these aggressive cancers that currently have no identified therapeutic targets that can be targeted for management. Finally, further studies are required to validate the efficacy of targeting FoxM1 expression with combination of other currently used chemotherapeutic agents for response and toxicity in BC.

## MATERIALS AND METHODS

### Patient selection and tissue microarray construction

One thousand and nine (1009) clinical samples from patients diagnosed with BC between 1990–2012 at King Faisal Specialist Hospital and Research Center (KFSHRC) were collected with complete clinical and histopathological data. All the collected formalin-fixed, paraffin-embedded blocks were processed for preparation of a TMA as described previously [45]. After TMA preparation, slides were prepared and manual staining was performed as described before [46]. Institutional Review Board (IRB) of the King Faisal Specialist Hospital & Research Centre approved the study (RAC # 2140 008 and 2110 025).

### Immunohistochemistry (IHC) Staining

Standard protocol was followed for IHC staining. For antigen retrieval, Dako (Dako Denmark A/S, Glostrup, Denmark) Target Retrieval Solution pH 9.0 (Catalog number S2368) was used, and the slides were microwaved at 750W for 5 min and then at 250W for 20 min. The primary antibodies used for staining tissue microarray sections and their dilutions are listed in Supplementary Table 1. The Dako Envision Plus System kit was used as the secondary detection system with 3, 30-diaminobenzidine as chromogen. All slides were counterstained with haematoxylin, dehydrated, cleared and mounted. Negative controls included omission of the primary antibody. Normal tissues of different organ system were also included in the TMA to serve as control. Only fresh cut slides were stained simultaneously to minimize the influence of slide aging and maximize reproducibility of the experiment.

### IHC scoring

Each TMA spot was assigned an intensity score from 0 to 3 (I0, I1–I3) and the proportion of tumor staining for that intensity was recorded as 5% increments from a range of 0–100 (P0, P1–P3). A final H score (range 0–300) was obtained by adding the sum of scores obtained for each intensity and proportion of area stained (H score = I1 × P1 + I2 × P2 + I3 × P3). FoxM1 was standardized, validated and scored as described earlier [40]. Using X-tile version 3.6.1 [40], we defined the optimal cut-off point for FoxM1, XIAP, Bcl-xL, VEGF and MMP-9. Based on X-tile plots, Breast cancer patients were sub-grouped into two groups, one with complete absence of staining (H score = 0) was defined as no expression; the other group with staining (H score > 0) was defined as over-expression for FoxM1. Similarly, X-tile was used to classify breast cancer patients into subgroups based on expression of Bcl-xL, XIAP, VEGF and MMP-9 as described previously [40].

### Cell culture

BC cell lines, CAL-120 and MDA-MB-231 were purchased from American Type Culture Collection (ATCC) and grown in RPMI1640 media supplemented with 10% FBS. Human umbilical vein endothelial cells (HUVECs) were purchased from ATCC and grown in EBM-2 (Lonza, Walkersville, MD). Cells were cultured at 37°C under a humidified 95%: 5% (v/v) mixture of air and CO_2_.

### Reagents and antibodies

Thiostrepton (FoxM1 selective inhibitor) was purchased from Tocris Cookson Inc (Ellisville, MO). Antibodies against caspase-3, caspase-9, p-AKT, Cox IV, β-actin, MMP-2, MMP-9, Bcl-2, BID, Bcl-xL and poly (ADP) ribose polymerase (PARP) antibodies were purchased from Cell Signaling Technologies (Beverly, MA). FoxM1, VEGF, Cytochrome c and GAPDH antibodies were purchased from Santa Cruz Biotechnology, Inc. (Santa Cruz, CA). XIAP antibody and trans-well invasion and migration kits were purchased from BD Biosciences (San Jose, CA). Annexin V and JC1 were purchased from Thermo Fisher Scientific (Waltham, MA). Live dead assay kit was purchased from Life Technologies (Grand Island, NY).

### Cell viability MTT assay

BC cells were plated at a concentration of 10^4^ cells in 0.2 ml of media in a 96 well format for 24 hours. Following incubation, cells were treated with various concentrations of thiostrepton for 48 hours and cells were stained with MTT and analyzed on a colorimetric plate reader as described previously [40].

### Cell death and apoptosis analysis

BC cells were treated with different concentrations of thiostrepton for 48 hours and analyzed for cell death and apoptosis. For cell death, following treatment, cells were stained with Calcein and Ethidium homodimers as described before [47]. The cells were then examined under a microscope with long pass filter. For cell cycle analysis and apoptosis, cells were either stained with Propidium iodide alone for cell cycle or annexin V and Propidium iodide for apoptosis and analyzed by flow cytometry as previously described [48].

### Cell lysis and immunoblotting

Following treatment, BC cells were lysed in phosphorylation lysis buffer containing 50 mM Hepes (pH 7.3), 150 mM NaCl, 1.5 mM MgCl_2_, 1.0 mM EDTA (pH 8.0), 100 mM NaF, 10 mM Na_2_H_2_P_2_O_7_, 200 µM Na_3_VO_4_ and 1X proteasome inhibitors (Roche pharmaceuticals, Basel, Switzerland). Following lysis, cells were spun at 14,000 rpm for 15 minutes at 4°C and protein amounts were measured using Bradford assay (Life Technologies). Equal amounts of protein were separated on SDS-Page and immunoblotted with different antibodies as described previously [49].

### Gene silencing using siRNA

Non-specific control siRNA and FoxM1 specific siRNA were used to transfect BC cells using Lipofectamine 2000 (Invitrogen, Carlsbad, CA) as described by the manufacturer. Following transfection, cells were harvested and proteins were isolated and immunoblotted with different antibodies.

### Measurement of mitochondrial membrane potential and cytochrome c release

Following treatment with thiostrepton for 48 hours, BC cells were harvested and stained with JC1 dye for 30 minutes at 37°C and analyzed by flow cytometry. In the same experiment, cells were also fractionized into mitochondrial free cytosolic and cytosolic free mitochondrial fractions and separated on SDS-Page for immunoblotting as described previously [40, 50].

### Soft agar colony assays

BC cells were treated with thiostrepton for 48 hours and then plated in 0.5 ml culture medium containing 0.4% (v/v) top agar and 20% FBS layered over a basal layer of 0.8% (v/v) agar and 20% FBS. The cells were then incubated at 37^o^ C in 5% CO_2_ for 4 weeks as described previously [14]. After 4 weeks incubation, cells were stained with staining solution (EMD Millipore, Billerica, MA) and visualized under an inverted microscope.

### Cell invasion and migration assays

Cell invasion and migration assay were performed as described previously [26]. Briefly, after treatment of cells with thiostrepton for 48 hours, cells were re-counted and equal number of cells were seeded into Trans-well inserts either uncoated (for migration assay) or coated (for invasion assay) with growth factor-reduced matrigel for 24 hours. After incubation, cells were stained with Diff-Quick stain set (Fisher Scientific, Pittsburg, PA), and photographed under a fluorescent microscope.

### Wound-healing migration assay

HUVECs were grown into wells of collagen coated 24 well plate dishes to 100% confluence. Cells were starved to inactivate cell proliferation and then wounded by 200 μl pipette tips. EBM-2 containing 0.5% FBS was added along with conditioned medium collected from BC cells with or without thiostrepton treatments. Images of the cells were taken after 24 h of incubation. The wound area was measured using Image J software, and data was expressed as percentage of open wound area calculated by dividing migrated distance by scratched distance. Three independent experiments were performed.

### Capillary-like tube formation assay

HUVECs in EBM-2 medium were seeded into the matrigel layer in 24–well plates at a density of 6x10^4^ cells/well. Conditioned media from thiostrepton treated and untreated BCs cells were added into the wells and incubated for 24 h at 37°C in 5% CO_2_ atmosphere. Tube formation was examined and photographed using an inverted microscope (20X) [35].

### Immunofluorescence analysis

BC cells grown on coverslips in 6-well plates were fixed with ice-cold 100% methanol followed by permeabilization with 0.2% Triton X-100, blocked with 5% horse serum in PBS solution, and incubated with antibodies to FoxM1 (sc-376471, 1:100) and VEGF (ab46154, 1:200) in buffer A (1% BSA, 0.1% Triton X-100, 10% horse serum in PBS solution) for 1 h at 37 °C. Cells were then incubated with Alexa Fluor 488 goat anti-mouse or Alexa Fluor 594 goat anti-rabbit antibody and mounted using DAPI. The cells were visualized using Olympus BX63 fluorescence microscope.

### Chromatin immunoprecipitation (ChIP) assay

ChIP analysis was performed using a Pierce TMA garose ChIP Kit (Thermo Scientific, Rockford, IL). Sheared chromatin was diluted and immunoprecipitated with 2 μg of anti-FoxM1 or control IgG antibody. DNA protein complexes were eluted from protein A/G agarose beads using a spin column and were reverse cross-linked by incubating with NaCl at 65°C. The intensity of FoxM1 binding to the VEGF promoter was analyzed by Applied Biosystems^®^ 7500 Fast Real-Time PCR Detection System with SYBR Green PCR master mix using following primer sequences, FoxM1 binding to the VEGF promoter sites, F1 (−1,635 to −1,420 bp): (F) GGAGCGTTTTGGTTAAATTGAG and (R) TGCATATAGGAAGCAGCTTGGA; F2 (−634 to −442 bp): (F) CCCCTTTCCAAAGCCCATTCC and (R) CCTTCTCCCCGCTCCAACACCC. General PCR amplification also performed in a Mastercycler® thermal cycler (Eppendorf, Foster City, CA).

### Animals and xenografts study

Six-weeks old female nude mice were obtained from Jackson Laboratories (Bar Harbor, ME) and maintained in a pathogen-free animal facility at least 1 week before use. All animal studies were done in accordance with institutional guidelines. For xenograft study, MDA-MB-231 cells (10 × 10^6^ cells per mouse) were resuspended in serum-free media (200 µl) and subcutaneously injected into the right abdominal quadrant of nude mice. After 1 week, mice were randomly segregated into three groups. Mice were then treated with two doses of thiostrepton 40 mg/kg/dose and 80 mg/kg/dose dissolved in DMSO intra peritoneal (i.p) twice weekly and vehicle DMSO-treated control groups, (*n* = 5). After 4 weeks of treatment, mice were sacrificed and tumors were collected. The body weight and tumor volume of each mouse was monitored weekly [40].

### Statistical analysis

Contingency table analysis and chi-square tests were used to study the relationship between clinico-pathological variables and FoxM1 expression. Survival curves were generated using the Kaplan-Meier method, with significance evaluated using the Mantel-Cox log-rank test. The limit of significance for all analyses was defined as a *p*-value < 0.05. The JMP 11.0 (SAS Institute, Inc., Cary, NC) software package was used for data analyses.

For all functional studies, data presented are means ± SD of triplicates in an independent experiment, which was repeated for at least two times with the same results. For multiple comparisons, one-way analysis of variance (ANOVA) was performed using IBM SPSS Statistics 21 software (IBM Corp., Armonk, NY). Values of *p* < 0.05 were considered statistically significant.

## SUPPLEMENTARY MATERIALS FIGURES AND TABLES


