# Supplementary material for: FoxM1 is an independent poor prognostic marker and therapeutic target for advanced Middle Eastern breast cancer
Source: Oncotarget. 2018 Apr 3;9(25):17466–82. doi: 10.18632/oncotarget.24739 (PMC5915129; doi:10.18632/oncotarget.24739)
Supplement: Supplementary file 1 [file oncotarget-09-17466-s001.pdf]

FoxM1 is an independent poor prognostic marker and therapeutic target for advanced Middle Eastern breast cancer

SUPPLEMENTARY MATERIALS

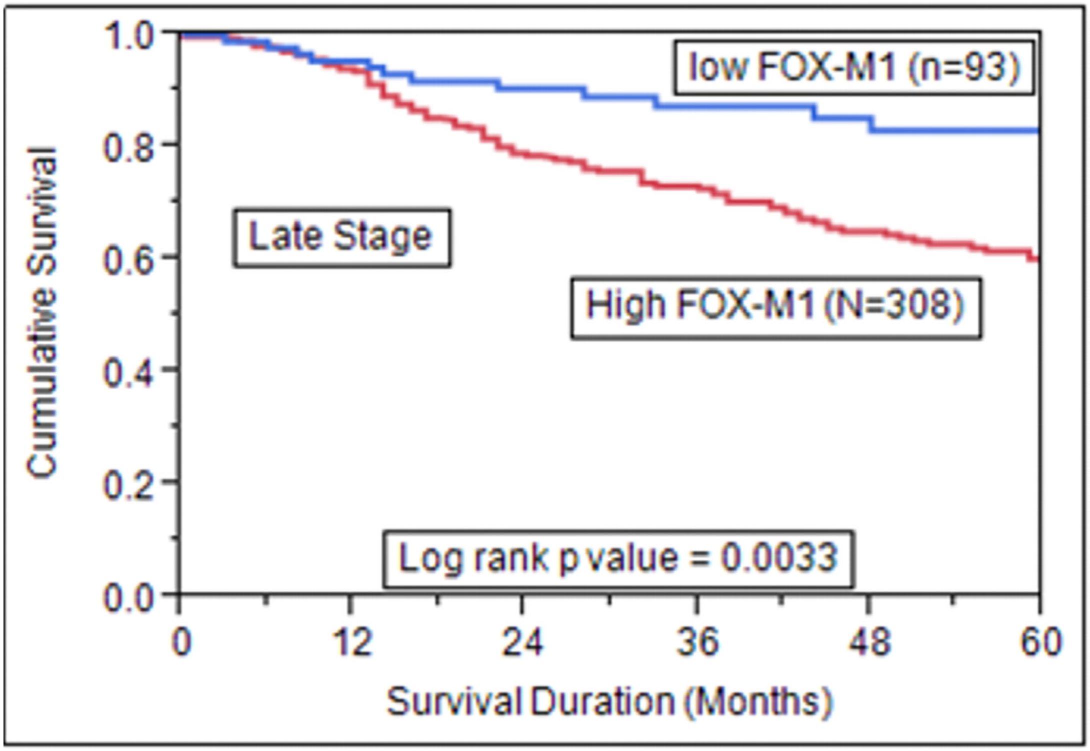

Supplementary Figure 1: Kaplan-Meier survival analysis for the prognostic significance of FoxM1 expression in late stage breast cancer. Breast cancer patients with overexpression of FoxM1 had reduced overall survival at 5 years compared to tumors showing low expression of FoxM1 in late stage ($p = 0.0033$).

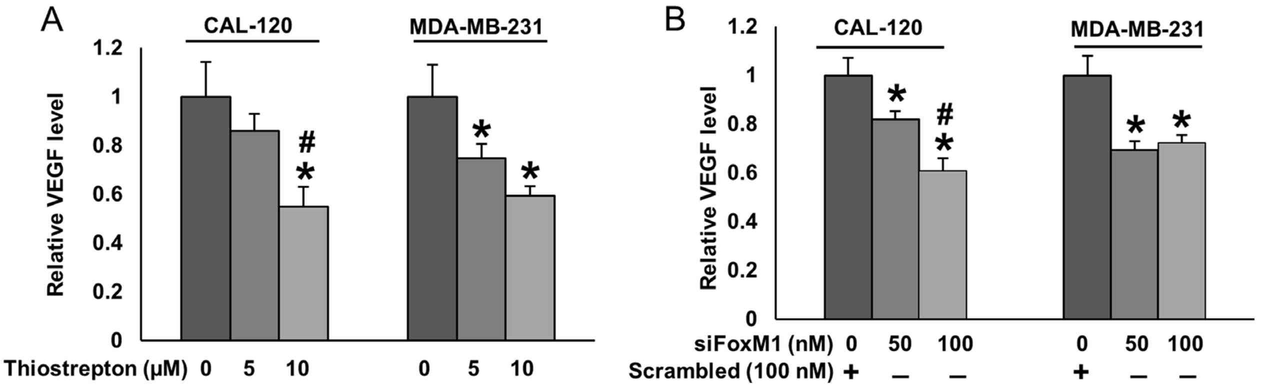

Supplementary Figure 2: (**A–B**) Inhibition of FoxM1 in BC cells decreased VEGF secretion in EOC cells. BC cells were treated with indicated doses of thiostrepton or FoxM1 siRNA for 48 hours, and secreted VEGF level in the media was estimated by VEGF ELISA kit (Thermo Fisher Scientific) according to the manufacturers' recommendations. Data presented in bar graphs are the mean ± SD of three independent experiments. *and # indicate statistically significant differences compared to control/scramble siRNA without treatment or thiostrepton (5 µM)/siFoxM1 (50 nM) treatment, respectively with $p < 0.05$.

Supplementary Table 1: Details of primary antibodies used in the study

Antibody	Clone	Company	Dilution	Retrieval	Detection
FoxM1	*polyclonal*	SantaCruz	1:2500	pH6, pressure cooker	Envision+
Ki-67	MIB-1	DAKO	1:500	pH9, pressure cooker	Envision+
p-AKT(Ser473)	D9E	Cell Signaling	1:20	pH9, pressure cooker	Envision+
XIAP	48	BD	1:400	pH9, pressure cooker	Envision+
VEGFA	VG1	Novus	1:2000	pH6, pressure cooker	Envision+
BCL-XL	54H6	Cell Signaling	1:800	pH9, pressure cooker	Envision+
MMP-9	54-2A4	Calbiochem	1:2000	pH6, pressure cooker	Envision+

Supplementary Table 2: Univariate and multivariate analysis of FoxM1 (only late stage cases) using cox proportional hazard model

Clinical Parameters	UNIVARIATE		MULTIVARIATE	
	**Risk Ratio (95% CI)**	***p* value**	**Risk Ratio (95% CI)**	***p* value**
**Age**<br>Above > 30	0.86 (0.41–2.24)	0.7369	1.97 (0.55–3.10)	0.6751
**Grade**<br>Poorly Diff.	1.63 (1.15–2.32)	**0.0068**	1.29 (0.88–1.89)	0.1947
**Histology**	0.38 (0.12–0.89)	**0.0237**	0.38 (0.09–1.03)	0.0600
**Triple Negative BC**	2.35 (1.52–3.52)	**0.0002**	1.92 (1.20–3.00)	**0.0071**
**Fox-M1**	2.20 (1.32–3.92)	**0.0016**	1.82 (1.06–3.37)	**0.0298**
